# Supplementary material for: Carleman linearization approach for chemical kinetics integration toward quantum computation
Source: Sci Rep. 2023 Mar 9;13:3935. doi: 10.1038/s41598-023-31009-9 (PMC9998465; doi:10.1038/s41598-023-31009-9)
Supplement: Supplementary file 1 — Supplementary Information. [file 41598_2023_31009_MOESM1_ESM.docx]

## Appendix: The overview of the HHL algorithm

The HHL^1–3^ is the quantum algorithm to efficiently obtain the solution vector $\boldsymbol{x}$ of huge linear equations $A\boldsymbol{x}=\boldsymbol{b}$. Before explaining the algorithm, we would like to prepare some information.

1. Let $A$ be the Hermitian matrix (if not, general matrix can be made Hermitian by $\left( \begin{matrix} O & A \\ A^{\dagger} & O \end{matrix} \right)$.)
2. Let $\boldsymbol{u}_{i} and \lambda_{i}$ be the *i*-th eigenvectors and eigenvalues of matrix $A$.

The quantum diagram of HHL can be illustrated in Fig. A-1^4^.


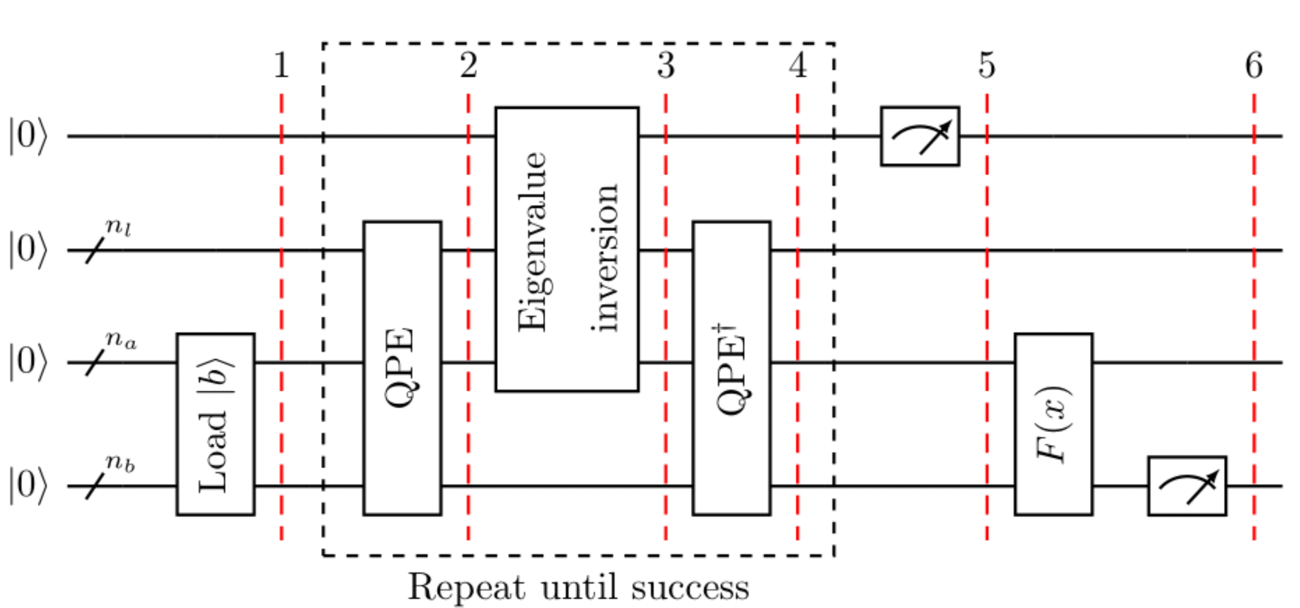


Fig. A- 1 The quantum diagram of HHL^4^.

Firstly, the vector $\boldsymbol{b}$ is prepared with the basis of matrix $A$ as follows:

|  | $\left\vert b \right\rangle=\Sigma_{j}\beta_{j}\left\vert u_{j} \right\rangle.$ | (A-) |
| --- | --- | --- |

Second, the quantum phase estimation (QPE) is applied for Eq. A-1. QPE is the algorithm to approximate the eigenvalues $exp(i\lambda_{j}t)$ of matrix $exp(iAt)$ as follows:

|  | $\left\vert0 \right\rangle_{j}\left\vert u_{j} \right\rangle⟼\left\vert\tilde{\lambda_{j}} \right\rangle\left\vert u_{j} \right\rangle.$ | (A-) |
| --- | --- | --- |

Where $\tilde{\lambda_{j}}$ is the binary expression of $\lambda_{j}$ estimated with *n*-qubits. By applying QPE for the input vector b, the state vector becomes

|  | $\sum_{j} \beta_{j}\left\vert\tilde{\lambda_{j}} \right\rangle\left\vert u_{j} \right\rangle$ | (A-) |
| --- | --- | --- |

Next step is rotation part. By performing the controlled *σ_y_*-rotation operation (supplemental control bit shown as *S*), the results of the Eq. A-3 is transformed to

|  | $\sum_{j} \beta_{j}\left\vert\tilde{\lambda_{j}} \right\rangle\left\vert u_{j} \right\rangle\left( \sqrt{1-\frac{1}{{\tilde{\lambda_{j}}}^{2}}}\left\vert0 \right\rangle_{S}+\frac{1}{\tilde{\lambda_{j}}}\left\vert1 \right\rangle_{S} \right).$ | (A-) |
| --- | --- | --- |

Finally, inverse of QPE is applied to Eq. A-4 and the state vector becomes

|  | $\sum_{j} \beta_{j}\left\vert0 \right\rangle_{j}\left\vert u_{j} \right\rangle\left( \sqrt{1-\frac{1}{{\tilde{\lambda_{j}}}^{2}}}\left\vert0 \right\rangle_{S}+\frac{1}{\tilde{\lambda_{j}}}\left\vert1 \right\rangle_{S} \right).$ | (A-) |
| --- | --- | --- |

By observing 1 for supplemental qubit ($\left| 1 \right\rangle_{S}$) with the supplemental clock bit 0 ($\left| 0 \right\rangle_{j}$), we obtain

|  | $\sum_{j} \beta_{j}\left\vert u_{j} \right\rangle\frac{1}{\tilde{\lambda_{j}}}$ | (A-) |
| --- | --- | --- |

Which is the solution of original problem ($\boldsymbol{x}=A^{-1}\boldsymbol{b=}\Sigma_{j}({\beta_{j}}/{\lambda_{j}})\boldsymbol{u}_{j}$).

## References in appendix

1. Harrow, A. W., Hassidim, A. & Lloyd, S. Quantum algorithm for linear systems of equations. *Phys. Rev. Lett.* **103**, 1–15 (2009).

2. Dervovic, D. *et al.* Quantum linear systems algorithms: a primer. *arXiv* 1802.08227 (2018).

3. Childs, A. M., Kothari, R. & Somma, R. D. Quantum Algorithm for Systems of Linear Equations with Exponentially Improved Dependence on Precision. *SIAM J. Comput.* **46**, 1920–1950 (2017).

4. Qiskit. Solving Linear Systems of Equations using HHL. *IBM* https://qiskit.org/textbook/ch-applications/hhl_tutorial.html.
